# Neurofilament light is a biomarker of brain involvement in lupus and primary Sjögren’s syndrome

**DOI:** 10.1007/s00415-020-10290-y

**Published:** 2020-10-30

**Authors:** Anne B. Tjensvoll, Maria B. Lauvsnes, Henrik Zetterberg, Jan T. Kvaløy, Ingeborg Kvivik, Stian S. Maroni, Ole J. Greve, Mona K. Beyer, Shunsei Hirohata, Chaim Putterman, Guido Alves, Erna Harboe, Kaj Blennow, Lasse G. Gøransson, Roald Omdal

**Affiliations:** 1 Department of Neurology, Stavanger University Hospital, Stavanger, Norway; 2 Department of Internal Medicine, Clinical Immunology Unit, Stavanger University Hospital, POB 8100, 4068 Stavanger, Norway; 3 Clinical Neurochemistry Laboratory, Sahlgrenska University Hospital, Mölndal, Sweden; 4 Department of Psychiatry and Neurochemistry, Institute of Neuroscience and Physiology, The Sahlgrenska Academy At the University of Gothenburg, Mölndal, Sweden; 5 UK Dementia Research Institute At UCL, London, UK; 6 Department of Neurodegenerative Disease, UCL Institute of Neurology, London, UK; 7 Research Department, Stavanger University Hospital, Stavanger, Norway; 8 Department of Mathematics and Physics, University of Stavanger, Stavanger, Norway; 9 Clinical Neuropsychology Unit, Division of Psychiatry, Stavanger University Hospital, Stavanger, Norway; 10 Department of Radiology, Stavanger University Hospital, Stavanger, Norway; 11 Institute of Clinical Medicine, University of Oslo, Oslo, Norway; 12 Division of Radiology and Nuclear Medicine, Oslo University Hospital, Oslo, Norway; 13 Department of Rheumatology and Infectious Diseases, Kitasato University School of Medicine, 1-15-1 Kitasato, Sagamihara, Kanagawa 252-0374 Japan; 14 Division of Rheumatology, Albert Einstein College of Medicine and Montefiore Medical Center, Bronx, NY USA; 15 Azrieli School of Medicine Bar-Ilan University, Zefat, Israel; 16 Galilee Medical Center Research Institute, Nahariya, Israel; 17 The Norwegian Centre for Movement Disorders and Department of Neurology, Stavanger University Hospital, Stavanger, Norway; 18 Department of Chemistry, Bioscience and Environmental Engineering, University of Stavanger, Stavanger, Norway; 19 Department of Clinical Science, Faculty of Medicine, University of Bergen, Bergen, Norway

**Keywords:** Neurofilament light chain, Anti-NR2 antibodies, Cognitive dysfunction, Systemic lupus erythematosus, Primary Sjögrens´s syndrome

## Abstract

**Background:**

To test the hypothesis that neurofilament light (NfL) in CSF is a biomarker of CNS involvement in patients with systemic lupus erythematosus (SLE) and primary Sjögren’s syndrome (pSS), we measured NfL in CSF from 52 patients with lupus and 54 with pSS and explored associations with clinical, structural, immunological and biochemical abnormalities.

**Methods:**

In CSF, we measured NfL, anti-P antibodies, protein S100B and TWEAK by ELISA and anti-NR2 antibodies by electrochemiluminescence. Anti-phospholipid antibodies and routine immunological tests were performed in blood. IgG and albumin were measured in CSF and serum for assessment of the blood–brain barrier function (Q-albumin) and intrathecal IgG production (IgG index). Cerebral MRI and neuropsychological testing were performed.

**Results:**

A multivariable regression model showed that increasing CSF anti-NR2 antibody levels were associated with increasing NfL levels in patients with SLE (*B* 1.27, 95% CI 0.88–1.65, *p* < 0.001). Age contributed significantly in the model (*B* 0.04, 95% CI 0.03–0.05, *p* < 0.001). Similar findings were observed in the pSS group. Adjusted for age and sex, no associations were found between NfL levels and any MRI data. In SLE patients, higher NfL concentrations were associated with impairments in psychomotor speed and motor function, and in pSS with motor dysfunction. These associations remained in multivariable regression models.

**Conclusions:**

Increased concentration of NfL in CSF is a marker of cerebral involvement in patients with SLE and pSS, is strongly associated with the presence of anti-NR2 antibodies, and correlates with cognitive impairment in several domains.

**Electronic supplementary material:**

**Supplementary information** is available for this paper at 10.1007/s00415-020-10290-y.

## Introduction

Systemic lupus erythematosus (SLE) is a chronic systemic inflammatory autoimmune disease that frequently involves the CNS [1]. The manifestations are diverse, varying from life-threatening strokes and encephalitis to headaches and mood disorders. Primary Sjögren’s syndrome (pSS) is another systemic inflammatory autoimmune disease that primarily attacks exocrine glands, such as the salivary and lacrimal glands, leading to dryness of the mouth and eyes [2]. Neuropsychiatric phenomena are common in both diseases [3].

**Fig. 1:**
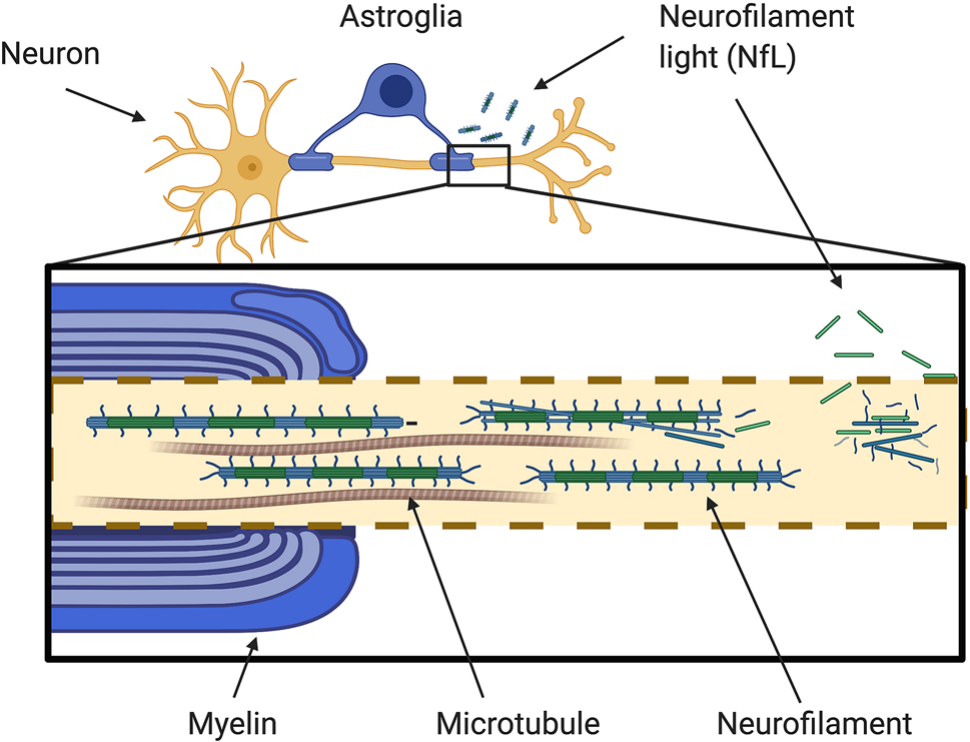
Neuron with neurofilaments. Figure illustrates disintegrating neurofilaments, and neurofilament light chains (NfL) leaking out of the axon. Figure created with BioRender.com

Neurofilament light chain protein (NfL) is one out of five neurofilament subunits that comprise the neuronal cytoskeleton. Increased NfL levels in CSF reflect axonal damage and degeneration; consequently, NfL is frequently used as a marker of CNS injury in neurodegenerative conditions, multiple sclerosis, cerebrovascular diseases, and traumatic brain injury (Fig. 1) [4]. We hypothesized that NfL levels in CSF would be increased in patients with SLE and/or pSS. We also hypothesized that increased NfL levels might be associated with brain reactive antibodies, such as anti-phospholipid antibodies (aPL antibodies) [5, 6], antibodies against the NR2 subunit of the *N*-methyl-d-aspartate receptor (anti-NR2 antibodies) [7, 8], or antibodies directed against ribosomal P proteins (anti-P antibodies) [9].

The present study aimed to determine whether CSF NfL could serve as a biomarker of CNS involvement in SLE and/or pSS. To that end, we investigated potential associations between NfL concentrations and structural, immunological, and biochemical abnormalities in patients with SLE and pSS. In addition, we investigated whether functional abnormalities were evident in patients with increased NfL levels, exemplified by cognitive dysfunction or headache.

## Methods

Nearly, all patients with systemic autoimmune diseases in Rogaland County, Norway, are allocated to Stavanger University Hospital, where this study was performed. Recruitment was based on hospital records from in- and outpatients.

### SLE group

Eighty-six patients, all Caucasian, fulfilled the 1982 revised American College of Rheumatology (ACR) criteria for SLE [10]. Of these, 70 (81%) consented to participate in the study. Two patients withdrew consent, and one was excluded, due to a brain tumor. Thus, 67 patients (78%) were included in the study.

### PSS group

Seventy-two (73%) out of 99 patients, all Caucasian, fulfilled the American European Consensus Group (AECG) criteria for pSS [11], and consented to participate in the study. One patient was excluded due to a brain tumor. Thus, 71 (72%) patients were included.

### Clinical examination

All patients were examined by two internists (EH and LG) and a neurologist (ABT) during a 2-day stay in the hospital, for research purposes only. SLE disease activity was assessed with the SLE disease activity index (SLEDAI) [12], and organ damage was assessed with The Systemic Lupus International Collaborating Clinics/American College of Rheumatology Damage Index (SLICC/ACR-DI) [13]. Headache was assessed in a structured interview, and classified according to the International Classification of Headache Disorders (ICHD II) [14]. Depression was assessed with the Beck Depression Inventory (BDI) applying a cut-off score of ≥ 13 to identify current clinical depression [15]. Fatigue severity was scored by the fatigue Visual Analogue Scale (fVAS) [16]. Arterial hypertension was defined as systolic blood pressure ≥ 140 and/or diastolic pressure ≥ 90 mmHg or current use of antihypertensive medication.

### Lumbar puncture

Fifty-two of the 67 patients with SLE (78%) and 54 of the 71 patients with pSS (76%) underwent lumbar punctures. All CSF samples were obtained between 1 and 2 p.m., placed on ice, and centrifuged at 4 °C at 3000×*g* for 10 min. Supernatants were immediately aliquoted and frozen at − 70 °C until analysis.

### Laboratory analyses

#### CSF

IgG was measured in CSF and serum with the Cobas Integra Immunoglobulin G (Turbidimetric) assay, and albumin with Tina-quant a Albumin Gen.2 (Roche Diagnostics, Mannheim, Germany) according to the manufacturer`s instructions. The CSF/serum albumin ratio (Q-albumin) was calculated as [CSF-albumin/serum-albumin] as a measure of the blood–brain barrier function, while the IgG index was calculated as [(CSF-IgG/serum-IgG)/(CSF-albumin/serum-albumin)] as a measure for intrathecal IgG production, and chronic CNS inflammation [17]. Anti-NR2 antibodies were detected by electrochemiluminescence [8] and anti-P antibodies by ELISA [18], both as previously described. Protein S100B was analyzed with the Human S100B ELISA kit (Abnova, Jhongli City, Taiwan) according to the manufacturer`s instructions, and TNF-like weak inducer of apoptosis (TWEAK) by ELISA (R&D Systems, Minneapolis, MN, USA) as previously described [19]. CSF NfL concentration was measured using a commercially available ELISA according to instructions from the manufacturer (UmanDiagnostics, Umeå, Sweden). The measurements were performed in one round of experiments by board-certified laboratory technicians who were blinded to the clinical data. Intra-assay coefficients of variation were below 10%.

#### Blood

Routine biochemical, hematological, and immunological analyses were performed at the hospital’s laboratories. ANA was detected with the HEp-2000 assay (Immunoconcepts, Sacramento, CA, USA), and presence of anti-double-stranded (ds) DNA by Nova Lite dsDNA Crithidia luciliae 708,200 indirect immunofluorescence assay (Nova Diagnostics, San Diego, CA, USA). Anti-SSA, and anti-SSB antibodies were measured by ELISA with QUANTA Lite ENA 6 assay (Inova Diagnostics, San Diego, CA, USA), and positive results confirmed by Quanta Lite SSA and SSB ELISA (Inova Diagnostics). Anti-cardiolipin IgM and IgG antibodies were measured with the QUANTA Lite™ ACA IgM and IgG ELISA (Inova Diagnostics). Lupus anticoagulant was measured by the activated partial thromboplastin time and dilute Russell’s viper venom time (Dade Behring, Marburg, Germany). Anti-phospholipid (aPL) antibodies were considered present if the patient had a positive anti-cardiolipin IgM- or IgG-antibody test, was lupus-anticoagulant positive, or any combinations of these.

#### MRI

MRI examinations were performed with a 1.5-T Philips Gyroscan NT Intera Release 10 (Philips Medical Systems, Best, The Netherlands). White matter hyperintensities (WMHs) were assessed in accordance with the semi-quantitative visual rating scale of Scheltens et al. [20]. Global GM and WM volumes were estimated using the VBM8 extension of the SPM8 software. Details of the MRI protocols and preprocessing are previously described [21].

### Neuropsychological testing

The tests were administered by a trained psychometric test technician. Results were analyzed by a clinical neuropsychologist (SSM). The test batteries included the Wechsler Memory Scale, Revised (WMS-R) [22], Wechsler Adult Intelligence Scale (WAIS) [23], Stroop Color-Word Interference Test [24], Wisconsin Card Sorting Test [25], FAS Verbal Influency Test [26], Tactual Performance Test (TPT), Fingertapping test, Trail Making Test A and B, Category Test, Seashore Rhythm Test, Lafayette’s Hand Dynamometer Test, and the Lafayette Grooved Pegboard Test [27]. These tests mapped functions in eight cognitive domains: memory, psychomotor speed, visual–spatial processing, motor function, language, reasoning/problem solving, simple attention and complex attention. Scores were compared to normative data for each test, and cut-off score for abnormality defined by a standardized score ≥ 2SD from the reference mean. Cognitive dysfunction was defined as abnormality in one or more of these domains. The neuropsychological tests are based on normative data that are adjusted for age, sex and education.

### Statistics

Continuous data are reported as medians and ranges. Categorical data are reported as numbers and percentages. Differences between groups were evaluated with the chi-square test for categorical data, and the Mann–Whitney *U* test for continuous data. NfL measurements were log-transformed to achieve a less skewed distribution, more appropriate for use in the statistical analyses. Linear regression analyses were performed to examine potential explanatory variables for NfL in CSF. All regression analyses with NfL as response variable were corrected for age by including age as adjustment variable. In the analyses of demographic and clinical variables, the explanatory variables were disease duration, hypertension, education, SLICC-DI and SLEDAI.

In the analyses with laboratory data, explanatory variables were sex, anti-NR2 in blood, aPL antibodies in blood and the following variables measured in CSF: anti-NR2 antibodies, anti-P antibodies, TWEAK, protein S100B and IgG. We first tested one explanatory variable at a time, adjusted for age. Then, we ran backward model selection, and the final model is reported. MRI analyses were performed with WMHs, global GM and—WM as response variables, NfL as explanatory variable and sex and age as adjustment variables.

Logistic regression analyses were performed to examine whether NfL was associated with abnormal cognitive domain scores. We ran both univariable analyses with only logNfL as explanatory variable, and each of the domain scores (dichotomized into normal/abnormal) as response, and multivariable analyses with logNfL, anti-NR2 antibodies, anti-P antibodies, TWEAK, and protein S100B as explanatory variables. Similar analyses were performed with clinical variables such as headaches, depression, or fatigue as response variables. Correction for multiple testing was not performed. *P* values < 0.05 were considered significant.

## Results

Selected clinical, laboratory and imaging data are shown in Table 1. SLE patients were younger than pSS patients. There were more cerebral infarcts in the SLE group, probably reflecting the higher prevalence of aPL antibodies compared with pSS patients. The pSS patients had lower mood than patients with SLE.

**Table 1:** Selected demographic and clinical data in SLE- and pSS patients

	SLE (*n* = 67)	pSS (*n* = 71)	*p* Value
Women/men, *n* (%)	58/9 (87/13)	61/10 (86/14)	> 0.99
Age, years	42.4 (20–76)	58.1 (27–87)	< 0.001
Education, years	13 (7–20)	12 (7–20)	0.34
Disease duration, years	11.0 (1–32)	6.1 (0–24)	< 0.001
SLEDAI scores	2.0 (0–26)	NA	NA
ANA positive, *n* (%)	65 (97)	59 (83)	0.007
Anti-SSA/SSB positive, *n* (%)	22 (33)	56 (79)	< 0.001
aPL positive, *n* (%)	26 (39)	9 (13)	0.001
BDI score	6.0 (0–27)	9.0 (0–38)	0.03
Fatigue VAS score	49 (1–98)	65 (3–96)	0.07
Arterial hypertension, *n* (%)	34 (51)	43 (61)	0.25
Current medications			
Corticosteroids, *n* (%)	44 (66)	16 (23)	< 0.001
Antimalarials, *n* (%)	33 (49)	26 (37)	0.13
MRI findings			
Cortical infarcts, *n* (%)	7/62 (11)	0/68	0.005
Lacunar infarcts, *n* (%)	8/62 (13)	2/68 (3)	0.05

Continuous data reported as median and ranges. Categorical data reported as numbers and percentages. The Mann–Whitney test was used to test for differences between the groups for continuous variables and the chi-square test for categorical variables

*SLE* systemic lupus erythematosus, *pSS* primary Sjögren`s syndrome, *SLEDAI* SLE disease activity index, *ANA* antinuclear antibodies, *SSA* Sjögren`s syndrome A antigen, *SSB* Sjögren`s syndrome B antigen, *aPL* anti-phospholipid antibodies, *BDI* Beck Depression Inventory, *VAS* visual analog scale

### NfL in CSF and demographic and clinical variables

**Fig. 2:**
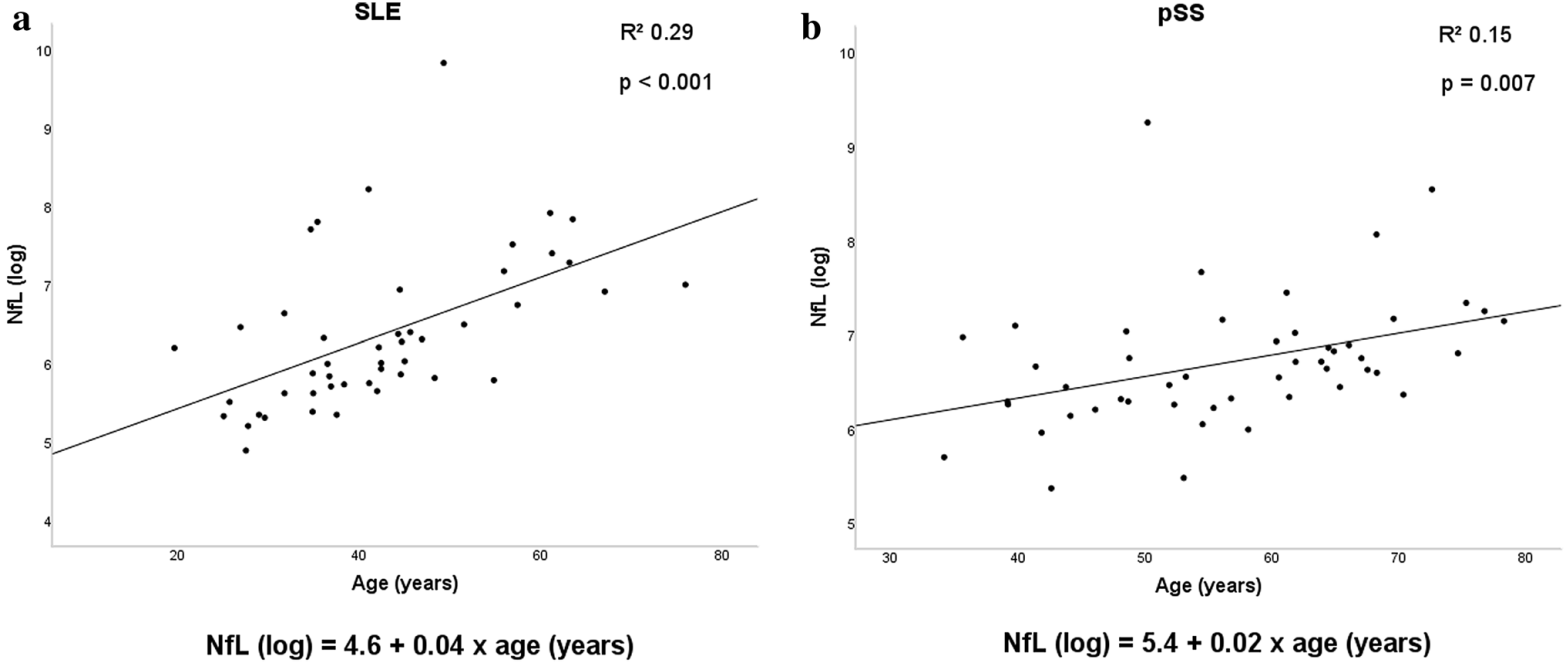
Influence of age on CSF NfL concentration. Results are shown for patients with (**a**) systemic lupus erythematosus (*N* = 47) and (**b**) primary Sjögren’s syndrome (*N* = 49)

CSF NfL levels were higher in pSS than SLE patients (Table 2), but after adjusting for age by a linear regression analysis, no significant difference in NfL between groups remained. The NfL levels increased with increasing age in both SLE (*B* 0.003, 95% CI 0.002–0.005, *p* < 0.001) and pSS patients (*B* 0.002, 95% CI 0.001–0.003, *p* = 0.007) (Fig. 2). Sex, disease duration or education did not influence NfL levels, neither in SLE patients nor in pSS patients. No associations were found between NfL levels and SLEDAI or SLICC-DI scores in the SLE patients (data not shown).

**Table 2:** CSF analyses

	SLE (*n* = 67)	pSS (*n* = 71)	*p* Value
NfL pg/mL	492 (133–18,608) (*n* = 47)	764 (214–10,439) (*n* = 49)	0.008
Anti-NR2 abª	0.38 (0.1–2.2) (*n* = 52)	0.41 (0.2–3.0) (*n* = 54)	0.366
Anti-P ab ug/mL	< 0.001 (< 0.001–0.13) (*n* = 51)	< 0.001 (< 0.001–0.04) (*n* = 54)	0.119
S100B pg/mL	222 (109.5–419.8) (*n* = 50)	264 (137.8–544.3) (*n* = 54)	0.003
TWEAK pg/mL	887 (369–2351) (*n* = 50)	1351 (481–5184) (*n* = 52)	< 0.001
IgG index	0.53 (0.45–1.57) (*n* = 52)	0.52 (0.41–2.05) (*n* = 54)	0.175

Data reported as median and ranges. P values calculated by the Mann–Whitney test

*SLE* systemic lupus erythematosus, *pSS* primary Sjögren`s syndrome, *NfL* neurofilament light, *ab* antibody

ªvalue given as a ratio of signal against an internal calibrator with defined signal intensity; TWEAK, TNF-like weak inducer of apoptosis

NfL levels were not associated with hypertension, fVAS scores, BDI scores, headaches in general or migraines in particular in either patient group (data not shown). In pSS patients, tension type headache was associated with higher NfL concentrations (OR 0.25, *p* = 0.02).

### NfL and other laboratory data

Measures of anti-NR2- and anti-P antibodies, protein S100B, TWEAK and IgG index in CSF are shown in Table 2. S100B and TWEAK were higher in pSS patients compared with SLE, but adjusted for age, only the difference for TWEAK remained (*B* 453.2, 95% CI 80.5–825.9, *p* = 0.02).

**Fig. 3:**
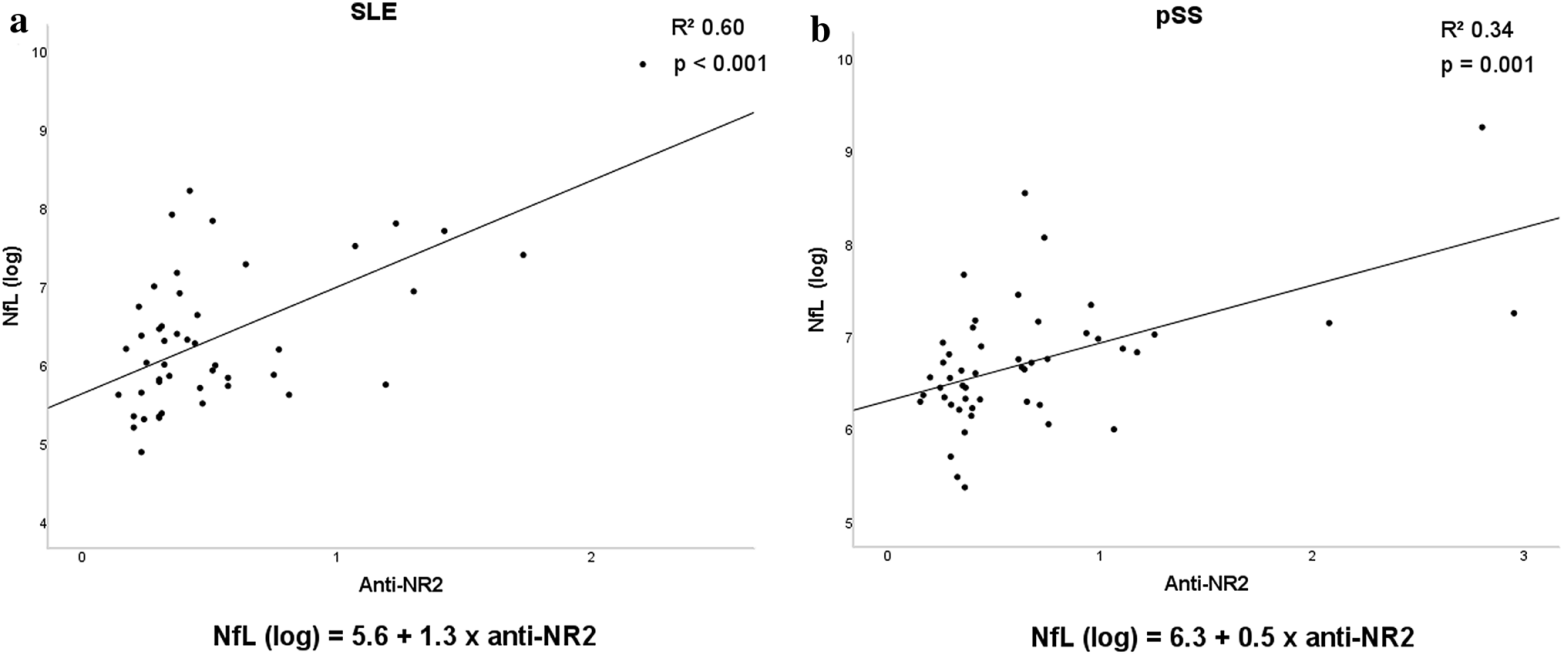
Associations between NfL and anti-NR2 antibodies in CSF. Results are shown for patients with (**a**) systemic lupus erythematosus (*N* = 47) and (**b**) primary Sjögren’s syndrome (*N* = 49)

In regression analyses, with NfL as response variable, we found that NfL levels increased with increasing CSF concentrations of anti-NR2 antibodies in both SLE (*B* 1.26, 95% CI 0.83–1.69, *p* < 0.001) and pSS patients (*B* 0.54, 95% CI 0.24–0.84, *p* = 0.001) (Fig. 3). No associations were revealed between anti-NR2 antibodies in blood and NfL in CSF in SLE (*B* − 0.11, 95% CI − 0.45–0.23, *p* = 0.53) or pSS patients (*B* 0.09, 95% CI − 0.16–0.34, *p* = 0.48).

Increasing levels of NfL were associated with increasing CSF concentrations of anti-P antibodies in pSS patients (*B* 27.0, 95% CI 10.6–43.3, *p* = 0.002), but not in SLE (*B* 11.6, 95% CI – 2.4–25.7, *p* = 0.10).

Furthermore, no association was revealed between NfL and S100B in SLE patients (*B* 0.001, 95% CI − 0.003–0.006, *p* = 0.59), or in the pSS patients (*B* 0.001, 95% CI − 0.001–0.003, *p* = 0.56).

A positive association was estimated between NfL and TWEAK in pSS patients, although not statistically significant (*B* 0.00016, 95% CI − 0.000009–0.00033, *p* = 0.06). In the SLE patients, the association was not significant either (*B* 0.00048, 95% CI − 0.00016–0.0011, *p* = 0.14).

Intrathecal IgG increased with increasing NfL in SLE (*B* 0.02, 95% CI 0.004–0.042, *p* = 0.02), but not in pSS patients (*B* 0.004, 95% CI − 0.002–0.010, *p* = 0.16).

A positive association was estimated between Q-albumin and NfL levels in SLE, however, not statistically significant (*B* 0.10, 95% CI − 0.005–0.214, *p* = 0.06), while not in pSS patients (*B* 0.03, 95% CI − 0.074–0.130, *p* = 0.59).

No associations were revealed between NfL and ANA or IgG indices in the two patient groups (data not shown).

In a multivariable regression model with logNfL as response variable, age as adjustment variable, and sex, aPL in blood, and anti-NR2-, anti-P antibodies, S100 B, TWEAK and IgG in CSF as explanatory variables, increasing levels of anti-NR2 antibodies were associated with increasing levels of NfL in the SLE patients (*B* 1.27, 95% CI 0.88–1.65, *p* < 0.001). Age also contributed significantly in the model, while no contribution of sex, anti-P antibodies, S100B, IgG, TWEAK or aPL was found. Also in the pSS group, increasing NfL levels were associated with anti-NR2 antibodies in CSF (*B* 0.54, 95% CI 0.24–0.84, *p* = 0.001). Final models are shown in Supplementary Table 1.

### NfL and MRI

In these analyses, we used MRI data as response variable, and because both age and sex can influence cerebral volumes, these variables were included as adjustment variables in the regression analyses with NfL as explanatory variable. No associations were evident between NfL levels and WMHs, global GM-, WM-, or hippocampus volumes in SLE or pSS patients (data not shown).

### Neuropsychological tests

Cognitive dysfunction defined as dysfunction in one or more domains, was evident in 30 (45%) of the 67 SLE patients and 35 (49%) of the pSS patients, *p* = 0.60. No differences between SLE- and pSS patients were evident regarding cognitive dysfunction in the specific domains (data not shown).

### NfL and neuropsychological data

In SLE patients, higher NfL concentrations were associated with impairment in psychomotor speed and motor function (Table 3). The associations remained in multivariable regression models.

**Table 3:** Impact of increasing NfL levels in CSF on risk for cognitive dysfunction

	SLE		pSS	
	OR (95% CI)	*p* Value	OR (95%CI)	*p* Value
Memory	0.33 (0.07–1.63)	0.17	1.63 (0.58–4.65)	0.36
Psychomotor speed	3.57 (1.48–8.64)	0.005	1.76 (0.72–4.30)	0.21
Visual-spatial processing	1.35 (0.38–4.79)	0.65	3.75 (0.83–16.95)	0.09
Motor function	2.28 (1.09–4.76)	0.03	9.07 (1.84–44.84)	0.007
Language	1.06 (0.47–2.40)	0.89	0.94 (0.24–3.70)	0.92
Reasoning /problem solving	NA		NA	
Simple attention	0.54 (0.10–2.81)	0.47	NA	
Complex attention (executive)	1.8 (0.90–3.61)	0.10	0.68 (0.26–1.79)	0.43

The table shows the increase in risk for cognitive dysfunction related to a one unit increase in log-NfL for each function score and patient group

*CI* confidence interval, *OR* odds ratio, *NA* not analysed due to no abnormal values in analysis. NfL was log-transformed to achieve a less skewed distribution

In pSS patients, higher NfL levels were associated with impaired motor function in both univariable and multivariable regression models.

## Discussion

The main finding in this study was that increasing concentrations of NfL in CSF was associated with increasing levels of anti-NR2 antibodies in CSF, and was a marker of cognitive dysfunction in patients with SLE as well as in pSS. We also investigated a panel of other biomarkers for brain involvement, and although several associations between NfL and these biomarkers were evident, anti-NR2 antibodies was the dominating actor with a strong and consistent association to NfL levels evident both in univariate and multivariate models. These findings complement previous studies demonstrating the pathogenetic potential of anti-NR2 antibodies for cerebral dysfunction in SLE and pSS [21, 28], and indicate that increased NfL levels in CSF reflect neuronal damage or dysfunction. These observations extend current knowledge in neurodegenerative-, inflammatory-, cerebral vascular disease, and head traumas, and show that NfL also in the two systemic inflammatory autoimmune diseases, SLE and pSS, can be regarded as a general biomarker of harmful neuronal CNS processes [29].

Only one previous study has investigated NfL in patients with SLE [30]. The authors of that study reported higher NfL levels in CSF in patients compared with healthy subjects. In addition, the highest NfL concentrations were found in SLE patients with NP involvement, and levels were lower after treatment with cyclophosphamide.

SLE and pSS are distinct and different diseases, but share similarities, such as an autoimmune pathogenesis, systemic inflammation and CNS involvement. NfL lacks disease specificity, and can be detected in low concentrations in the CSF of healthy persons and increases with increasing age [4]. We found a strong association between increasing NfL concentrations and increasing age both in SLE and pSS patients, and also that NfL concentrations were higher in patients with pSS compared with SLE patients (Table 2). However, the pSS patients were older than the SLE patients, and when adjusted for age, there were no differences in NfL concentrations between the diseases. This indicates that the neuronal pathogenetic impact is more or less similar in the two diseases.

A number of other biomarkers in CSF and blood were studied. Of these, anti-P antibodies have been shown to exert a pathogenetic effect on the brain in several studies, being associated with psychosis and depression in SLE patients [18]. We found no convincing contribution of intrathecal anti-P antibodies to NfL elevations, possibly indicating that they do not exert a general neuronal destructive effect. On the other hand, none of our patients had psychosis and anti-P levels were generally low and possibly not of a pathogenetic type.

S100B is produced by activated astrocytes as response to damage, danger or other homeostatic disturbances and signal through RAGE and TLR-4 on microglia thus initiating proinflammatory cytokine production and increasing intrathecal immune activity [31, 32]. Increasing NfL levels were associated with increasing S100B levels in pSS, but not the SLE patients. This observation could point to some different mechanism for brain involvement in the two diseases, but remains speculative. The association disappeared in multivariable testing, again showing that anti-NR2 antibodies were the dominating pathogenetic actors.

Q-albumin—an indirect measure of blood–brain barrier integrity—is calculated by the ratio of CSF versus blood albumin [17]. Increased Q-albumin is seen when the blood–brain barrier is leaking, and albumin passes from blood into the CSF, followed by increased passage of other proteins, including antibodies, cytokines and other bioactive molecules. Disrupted integrity of the blood–brain barrier has lately been advocated as an important and necessary step for neuropsychiatric SLE to develop [33], although some have questioned this [19, 34]. In this study, there was a tendency towards increasing Q-albumin increased NfL levels in the SLE patients, but not pSS. In multivariate statistics, no effect of Q-albumin was seen. Anti-NR2 antibodies remained as the only operative factor for NfL concentrations indicating that at least for pathophysiological processes reflected in increased NfL, the blood–brain barrier is of less importance.

Eight patients, one with SLE and seven with pSS had elevated IgG indices, reflecting intrathecal IgG synthesis. However, no associations between IgG indices and NfL levels were observed.

TWEAK is a member of the TNF superfamily of cytokines and acts through the receptor Fn14. Both are present in the CNS on endothelial cells, perivascular astrocytes, neurons and microglia [35, 36]. The function is unclear, complex, and probably depends on the local pathophysiological conditions. Some animal studies indicate that TWEAK opens up the blood–brain barrier, but this has not been confirmed in human studies [19, 37]. We have hypothesized that TWEAK is produced in human SLE and pSS as response to immunological stress and works as a “neuroprotective” protein in the CNS [19]. In the present study, increased levels of TWEAK was only weakly associated with increasing NfL levels in pSS patients, and this association disappeared in multivariable statistics. This fits the hypothesis that TWEAK does not facilitate the action of brain reactive antibodies by opening the BBB, but could rather be engaged in cellular homeostasis during inflammation and cellular stress*.*

APL antibodies were measured in blood and there were higher concentrations in the SLE than the pSS patients, as expected. There were more cerebral infarcts in the SLE group, but aPL did not influence NfL levels, indicating that their presence did not exert a chronic pathogenetic effect on the cerebral neurons. This finding is in line with the understanding that cognitive dysfunction in patients with aPL antibodies is caused by cerebral infarcts, and not due to an anti-neuronal effect of the antibodies.

A limitation of the study is the cross-sectional design and lack of a control group and a longitudinal design. Furthermore, there was a relatively low number of participants reflecting that both diseases are relatively rare diseases. In addition, it is hard to obtain high numbers of CSF samples for research in general, and from patients with rare diseases in particular. Relatively low disease activity in the SLE patients could possibly have influenced the results. On the other side, most neuropsychiatric manifestations are immunological targeted to specific structures, and not dependent on high inflammatory activity. The applied methods for cerebral image analyses were probably not sensitive enough to pick up minor morphological changes that we and others previously have found to be associated with the presence of anti-NR2 antibodies. No explicit adjustment for multiple testing was done, and we thus acknowledge that some small *p* values might have been obtained by chance. In particular, *p* values close to 0.05 should be interpreted with caution.

Strengths of the study are unselected patients with well-defined diseases, comprehensive and systematic clinical examination, and sampling of CSF and blood under standard conditions.

We conclude that increased concentrations of NfL in CSF is a marker of brain involvement in patients with pSS as well as in SLE and is reflected in cognitive impairment in several domains. Anti-NR2 antibodies are probably the pathogenetic actors that lead to neuronal distortions. This study is exploratory, and the observations need to be replicated in a prospective, hypothesis-testing design.

## Supplementary information

Supplementary Information.

## Data Availability

The data that support the findings of this study are available from the corresponding author upon reasonable request.
