# Supplementary material for: Neurofilament light is a biomarker of brain involvement in lupus and primary Sjögren’s syndrome
Source: J Neurol. 2020 Oct 30;268(4):1385–94. doi: 10.1007/s00415-020-10290-y (PMC7990817; doi:10.1007/s00415-020-10290-y)
Supplement: Supplementary file 1 — Supplementary Information. [file 415_2020_10290_MOESM1_ESM.docx]

Supplementary Table 1. NfL and anti-NR2 in multivariable analyses, final steps

SLE

| Final Model | B (95%CI) | P-value |
| --- | --- | --- |
| Anti-NR2 ratio CSF | 1.27 (0.88-1.65) | < 0.001 |
| Age | 0.04 (0.03-0.05) | < 0.001 |

pSS

| Final Model | B (95%CI) | P-value |
| --- | --- | --- |
| Anti-NR2 ratio CSF | 0.54 (0.24-0.84) | 0.001 |
| Age | 0.015 (0.0004- 0.031) | 0.045 |
